# Commentary: Ultrasound-Guided Biopsy of Pleural-Based Pulmonary Lesions by Injection of Contrast-Enhancing Drugs

**DOI:** 10.3389/fphar.2020.00365

**Published:** 2020-04-14

**Authors:** Carla Maria Irene Quarato, Salvatore De Cosmo, Federica D'Agostino, Giulia Gaudiuso, Marco Sperandeo

**Affiliations:** ^1^ Department of Medical and Surgical Sciences, Institute of Respiratory Diseases, University of Foggia, Foggia, Italy; ^2^ Department of Internal Medicine, IRCCS Fondazione “Casa Sollievo Della Sofferenza”, Foggia, Italy; ^3^ Department of Medical and Surgical Sciences, Institute of Respiratory Diseases, University of Bari, Bary, Italy; ^4^ Department of Internal Medicine, Unit of Interventional and Diagnostic Ultrasound, IRCCS Fondazione “Casa Sollievo Della Sofferenza”, Foggia, Italy

**Keywords:** CEUS, US guided biopsy, lung, neoplasm cancer, diagnosis

Ying Fu et al. (2019) in the recent issue of this Journal published an interesting paper entitled “Ultrasound-Guided Biopsy of Pleural-Based Pulmonary Lesions by Injection of Contrast-Enhancing Drugs” on which we would like to express some views.

US-guided lung biopsy has success rate similar to CT-guided biopsies (which is the gold standard) in the assessment of the cyto-histological diagnosis of peripheral lung lesions, but offers lower complications, less time, and less cost without radiation exposure (Frongillo et al., 2018; Sperandeo et al., 2019). A new US contrast agent, SonoVue, (Bracco SpA), has emerged in recent years for US-guided biopsy of lesions of the liver (D'Onofrio et al., 2015), thyroid (Zhan and Ding, 2018), and mediastinal cancers (Fu et al., 2016), but it has also been proposed, off label, for the study of peripheral lung lesions in contact with the pleural surface (Cao et al., 2011; Dong et al., 2015; Sidhu et al., 2018).

In the paper, the authors stated that most malignant lung lesions “are supplied by the bronchial artery, so the time phase can be used to distinguish lesions originating from lung tissue or the bronchial blood supply, thereby avoiding an unnecessary needle biopsy.” However, as we well know from the study of anatomy, lungs have a dual blood supply that it is different from that of the liver in which we can identify an arterial, venous, and portal phase.

The pulmonary circulation begins at the main pulmonary artery and follows the system of airways to the alveolar capillaries. Here the mixed venous blood pumped by the right ventricle is oxygenated and returns to the left ventricle *via* the pulmonary veins. The bronchial circulation arises from the aorta and perfuses the entire lung parenchyma (with the exception of the alveoli) across the bronchial arteries. The venous blood of the bronchial circulation drains in part into the veins of the systemic circle that go to the right heart and, in a lesser part, into the pulmonary veins that run into the left heart, as a component of the physiological right-left shunt (West, 2012).

As a result, in the normal lung, after only 6-7 seconds from the infusion of the contrast, the four cardiac chambers will be completely perfused (i.e. both the pulmonary and systemic circulation will be perfused) (Nael et al., 2006). Therefore, the ecocontrastographic evaluation of lung subpleural lesions, after 7 seconds, reveals only the presence of vascularization but does not differentiate the pulmonary artery blood supply from the bronchial one.

In addition, as we know from physiology, in the upright human lung blood flow decreases almost linearly from bottom to top. This uneven distribution of blood flow can be explained by the intrapleural pressure gradient and gravity-related hydrostatic pressure differences between apical segments and those located at the base of the lung (West, 2012). Indeed, when the subject lies supine the distribution from apex to base becomes almost uniform, while blood flow in the posterior regions of the lung exceeds flow in the anterior parts. (MacNee et al., 1989).Variations in lung contrast-medium transit times can thus depend on the patient's position during the examination. Authors did not specify in what position their patients were examined (supine or seated).This seems important to standardize early and late phases of enhancement in CEUS studies of lung tumor.

Nevertheless, variations in transit time can be influenced by anastomoses between the two lung circulations as well as by the specific neovascularization characteristics of various types of lung tumors and the potential effects on pulmonary vascularization of concomitant cardiac or lung diseases (West, 2012), such as chronic obstructive pulmonary disease, pulmonary fibrosis, cardiopaty, atherosclerosis, hypertension, pneumoconiosis, etc.

Finally, authors claimed that CEUS can effectively make a distinction between necrotic (more often when size >5 cm) and active tissue. At this point, we should remember that the physiological response of the lungs to an hypoxic stimulus is a vascular reflex of constriction that leads to the redistribution of blood flow to better ventilated areas of lung (West, 2012). The same mechanism can be activated when hypoxia occurs for alveolar infarction given by neoplastic cells. This last condition represents, for example, a false negative for CEUS because it presents a falsely necrotic region of the lung. Therefore, the actual presence of necrosis inside the lesion should have been previously documented by a Chest TC scan.

In conclusion, in our opinion it is very important to emphasize the concept that CEUS cannot be used to differentiate a malignant lesion from a benign one due to the the particular anatomy and physiology of the dual blood supply of the lungs (Sperandeo et al., 2017).

Despite this, we agree that CEUS better highlights the vascularization of peripheral lung lesions and may be useful for increasing the accuracy of the biopsy for those lesions where the rate of necrosis, previously assessed on TC scan, is more than 50%. With this exception, our everyday life experience did not show a statistically significant difference between biopsies made with and without contrast medium (Del Colle et al., 2019). This is probably due to the fact that the diagnostic accuracy improves when the operator is an expert and during the execution of the biopsy exam he notices the lack of hardness of the pulmonary lesion and therefore makes several passes with the needle in a single session. The goal is to obtain adequate tissue once the material has been transferred to a glass slide. In addition, in order to improve the histologic diagnosis and minimize the occurrence of complications, we use a dedicated probe with a central hole and the “modified Menghini” technique (Sperandeo et al., 2014). It consists of the use of a needle with a Menghini type tip and a pyramidal stylet connected to a syringe plunger. The needle (18 gauge) is labeled with a centimeter scale to have the highest precision level. (Sperandeo et al., 2019) (**Figure 1**). Also, the pathologist's experience with pulmonary lesions is crucial for a satisfactory diagnosis.

**Figure 1:**
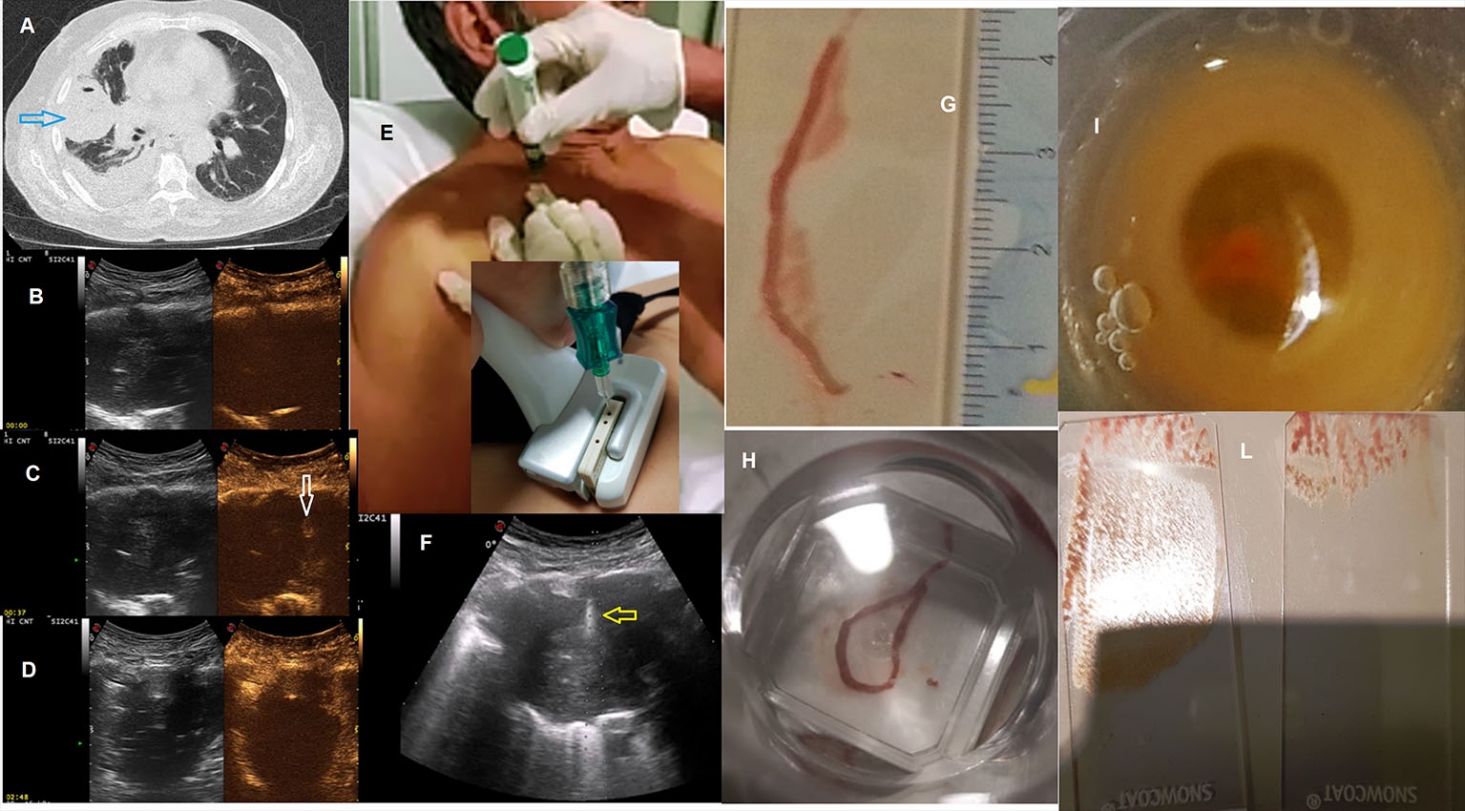
**(A)** Axial chest computed tomographic (CT) image detected a solid lung nodule suggestive of malignancy in the periphery of the right middle lobe (blue arrow) with pleural effusion. This lesion has broad pleural contact. Subsequent frames of contrast-enhanced ultrasound (CEUS) showing progression of enhancement followed **(B)** at start (0) **(C)** at 37 and **(D)** at 168 second; In **(C)** minimal enhancement of the lesion (white arrow) at 37 second. Metodology of Ultrasound guided biopsy. We can see: **(E)** seated position of the patient; **(F)** the needle (yellow arrow) within the consolidation in the right middle lobe. A dedicated probe with a central hole through which the needle set is introduced. Specimen suitable for histologic and cytologic diagnosis (adenocarcinoma, ALK positive): **(G, H)** biopsy, **(I)** cell block, **(L)** glass slides All the images included in this figure are original and never published before.

## Author Contributions

MS and CQ contributed to the conception and design of the study, to acquisition and interpretation of data, to drafting the work and revising it critically. FD'A, SD, and GG contributed to acquisition and interpretation of data, to drafting the work and revising it critically. All author have read and approved the final version of the paper.

## Conflict of Interest

The authors declare that the research was conducted in the absence of any commercial or financial relationships that could be construed as a potential conflict of interest.
